# Protection against cisplatin-induced acute kidney injury by hellgrammite extract: insights from comprehensive metabolomics

**DOI:** 10.3389/fphar.2026.1839763

**Published:** 2026-07-09

**Authors:** Kexin Lin, Guangling Lu, Rui Liu, Yanmei He, Xiaoyan Yuan, Xiaofei Li, Yiqin Wu, Jianyong Zhang, Chengquan Cao

**Affiliations:** 1 Yixing Center for Disease Control and Prevention, Wuxi, Jiangsu, China; 2 School of Pharmacy, Zunyi Medical University, Zunyi, China; 3 College of Life Sciences, Leshan Normal University, Leshan, China

**Keywords:** acute kidney injury, cisplatin, hellgrammite, metabolomics, traditional pharmacology

## Abstract

**Background:**

Cisplatin (CP), a highly potent antineoplastic agent, is severely limited in clinical use by dose-limiting toxicities, particularly acute kidney injury (AKI**)**. The hellgrammite (Hel), a kind of rare edible and medicinal insect, is known in Traditional Chinese Medicine (TCM) for its ability to tonify the qi and kidneys. However, its potential to ameliorate CP-induced AKI remains unexplored.

**Methods:**

We first evaluated the nutritional profile of Hel using high-performance liquid chromatography (HPLC) for amino acid composition analysis. Subsequently, a mouse model of CP-induced AKI was treated with different doses of Hel by gavage, then traditional pharmacology, and metabolomics were used to elucidate the effects and mechanism of Hel on CP-induced AKI mice.

**Results:**

Hel effectively ameliorated CP-induced alterations in biochemical indicators and alleviated kidney pathological changes. Hel administration rectified the kidney metabolic disturbances induced by CP, involving alterations in 38 differential metabolites associated with glycerophospholipid metabolism, purine metabolism, nicotinate and nicotinamide metabolism, and thiamine metabolism.

**Discussion:**

Hel confers renoprotection by reversing metabolic disorders in CP-induced AKI, revealing actionable targets for AKI intervention.

## 1 Introduction

Drug-induced acute kidney injury (AKI) represents a significant clinical burden due to its marked contribution to the morbidity and mortality of affected patients (Garcia et al., 2024). Cisplatin (CP) acts as a highly potent chemotherapeutic agent and as a first-line treatment for numerous solid tumors (Brown et al., 2019). However, its nephrotoxicity also makes it one of the leading causes of chemotherapy-associated AKI (Aati et al., 2025; Gao et al., 2020). Studies have reported that approximately 30% of patients develop AKI after a single dose of CP (50–100 mg/m^2^) (Mehta et al., 2016; Yang et al., 2018). CP primarily accumulates in the proximal tubular epithelial cells of the kidney, triggering inflammatory responses, cellular damage, and apoptosis, leading to tubular injury and tubulointerstitial nephritis (Gao et al., 2024). Current clinical treatments to prevent CP-induced AKI consists mainly of hydration, diuretics, and osmotic diuretics (Chen et al., 2022). However, the lack of effective preventive or therapeutic strategies often leads to dose limitations and increased patient risk.

Traditional Chinese Medicine (TCM) offers a distinct approach to AKI prevention and treatment due to its multi-target, multi-pathway characteristics and high clinical applicability (Chen et al., 2025; Wang et al., 2025). Research findings have indicated that many Chinese medicines and their active ingredients, such as icariin (Liu et al., 2024), ginkgo biloba extract (Zhang et al., 2023b), wogonin (Meng et al., 2018), leonurine (Hu et al., 2022), and astragaloside IV (Song et al., 2021), have shown protective effects against CP-induced AKI. Hellgrammite (Hel), the larva of Acanthacorydalis (Megaloptera: Corydalidae), has been used in TCM to tonify the kidneys, boost qi, and reduce urination, making it applicable for conditions such as nocturia and spermatorrhea due to kidney deficiency (Cao, 2014; Guo et al., 2012). Modern nutritional analysis indicates that Hel is rich in proteins, amino acids, polysaccharides, unsaturated fatty acids, and various minerals, contributing to its anti-inflammatory and antioxidant activities (Liu, 2019). Currently, studies have reported that Hel extract could promote growth and reproductive capability in *Drosophila melangogasters* and mice (Yang et al., 2014; Yang et al., 2012), while Hel plays a crucial role in urine concentration through the activation of AQP-2 and AVPR-V2 expression, thereby strengthening the contractile capacity of the kidney collecting ducts and promoting water reabsorption in the kidney tubules, resulting in an antidiuretic effect (Shi et al., 2019). Thus, Hel likely protects against CP-induced AKI by maintaining renal tubular structural integrity and function, though its precise protective efficacy and the underlying molecular mechanisms await further clarification.Metabolomics is a robust systems biology approach that employs advanced high-throughput analytical techniques to systematically characterize small-molecule metabolites within biological systems (Lin et al., 2024). By comprehensively profiling these metabolic changes, it can be utilized to characterize physiological or pathological states, assess the disruption of metabolic pathways, and identify sensitive biomarkers and potential therapeutic targets (Qiu et al., 2023). Consequently, metabolomics is highly beneficial for clarifying the connotation of TCM theories and holds considerable promise in elucidating complex therapeutic mechanisms and evaluating drug toxicity (Zhao et al., 2024). Building on this approach, our research group has previously utilized comprehensive metabolic profiling to successfully elucidate the underlying mechanisms of cantharidin-induced kidney injury and hepatotoxicity (He et al., 2023; Liu et al., 2020). However, studies evaluating the metabolic reprogramming induced by the medicinal insect Hel in the treatment of CP-induced AKI models remain scarce.This study employs a combined approach of metabolomics and metabolome-based pharmacology to investigate the protective effects and metabolic regulatory mechanisms of Hel-derived proteins in a CP-induced AKI mouse model. Firstly, high-performance liquid chromatography (HPLC) technology was used to systematically identify the types and concentrations of amino acids within the Hel, providing foundational data to comprehensively evaluate its nutritional value and support its development and utilisation. Subsequently, using the CP-induced AKI mouse model, we comprehensively validated the *in vivo* nephroprotective efficacy of Hel treatment. Finally, in-depth metabolomics profiling was performed to decode the metabolic reprogramming induced by Hel. By identifying the key metabolic pathways responsible for restoring renal homeostasis, this study provides a robust mechanistic basis for its therapeutic potential.

## 2 Materials and methods

### 2.1 Materials and chemicals

CP (FA1A2005A) was sourced from Qilu Pharmaceutical Co., Ltd., (Jinan, China). The Hel specimens were authenticated by Dr. Chengquan Cao from the field ecological breeding base in Yunnan Province, China. The species is not listed in CITES, and all material was collected and used domestically within China in compliance with the Nagoya Protocol. N-Butyl alcohol (20210808) was acquired from Tianjin Fuyu Fine Chemical Co., Ltd., (Tianjin, China).

### 2.2 Preparation and quality assessment of Hel crude extract

#### 2.2.1 Preparation of Hel extract

Hel was pulverized using an electric pulverizer to obtain Hel powder. The Hel powder (600 g) was immersed in methanol (6 L) for 24 h. The filtrate collected after filtration was concentrated via rotary evaporation to prepare the crude methanol extract, followed by three rounds of reflux extraction with n-butyl alcohol at 60 °C for 8 h per extraction. The combined extracts were dried to obtain Hel extract powder. Hel extract (4.5 g) was obtained ultimately, with an extraction rate of 0.75% attained.

#### 2.2.2 Quality analysis of Hel extract

Separation was performed on an Agilent InfinityLab Poroshell 120 HILIC-Z column (2.1 × 100 mm, 2.7 μm) at 40 °C. Mobile phase A was 20 mmol/L ammonium formate aqueous solution (pH = 3), and phase B consisted of 20 mmol/L ammonium formate aqueous solution (pH = 3) in acetonitrile/water (9:1, v/v). The injection volume was 0.5 μL with a flow rate of 0.4 mL/min, and the gradient program was: 0–11.5 min (100% B), 11.5–11.6 min (100%–70% B), 11.6–15 min (40% B). Mass spectrometric (MS) detection was conducted using an ESI source in MRM mode, with the following parameters: capillary voltage 1500 V; drying gas (330 °C, 13.0 L/min); nebulizer 35 psi; sheath gas (390 °C, 12 L/min). The qualitative and quantitative analytical results of 21 amino acids were summarized in Table 1.

**TABLE 1:** 21 amino acid contents of hellgrammite.

No	Name	RT/min	Parent ion (m/z)	Quantitative ion (m/z)	Collision energy/V	Qualitative ion (m/z)	Collision energy/V	Fragmentation voltage/V	Content (ng/mL)
1	Phenylalanine	3.42	166.1	120.1	13	103.0	29	80	2,482.09
2	Tryptophan	3.56	205.1	188.0	8	146.0	20	80	12.24
3	Soleucine	3.82	132.1	86.1	9	44.2	25	75	8,026.13
4	Leucine	3.82	132.1	86.1	9	30.2	17	75	71.40
5	Hydroxyproline	4.10	132.1	86.1	16	68.1	24	80	94.04
6	Tyrosine	4.82	182.1	136.1	13	91.1	33	85	3,204.99
7	Valine	5.09	118.1	72.1	9	55.1	25	70	2,189.28
8	Proline	5.24	116.1	70.1	17	43.2	37	75	—
9	Alanine	5.75	90.1	45.0	40	44.2	9	70	3,927.48
10	Serine	5.85	120.1	74.1	9	56.1	17	75	5,757.86
11	Butyric acid	5.95	104.1	87.1	8	69.1	16	70	7.63
12	Glycine	6.21	76.0	30.1	12	—	—	60	7,834.16
13	Glutamine	6.38	147.1	130.1	9	84.1	17	80	—
14	Threonine	6.40	106.1	88.1	8	42.2	24	67	5,630.38
15	Citrulline	7.02	176.1	159.1	9	70.1	25	80	70.79
16	Histidine	7.47	156.1	110.1	13	83.1	29	90	635.11
17	Glutamate	7.75	148.1	130.0	5	84.1	17	75	8,287.17
18	Aspartate	8.14	134.0	88.1	9	74.0	13	70	13,018.89
19	Arginine	8.77	175.1	70.1	24	60.1	12	100	1,558.56
20	Lysine	9.31	147.1	130.1	9	84.1	17	75	9,651.29
21	Asparagine	9.43	133.1	116.1	8	70.1	20	70	983.32

### 2.3 Animal experiments and drug interventions

Male ICR mice (20–22 g) were acquired from Hangzhou Ziyuan Laboratory Animal Technology Co., Ltd. (SCXK2019-0004) and housed in a controlled environment (50% ± 5% humidity, 22 °C ± 2 °C, and 12 h light/dark cycles) with unrestricted access to food and water. After a one-week acclimation period, the mice were randomly divided into five experimental groups (n = 12 in each group): Control, CP, CP+Hel-L, CP+Hel-H, and Hel-H only group. We determined the administration dosage of Hel extract by integrating traditional use practices with published literature (Shi et al., 2019). Mice in the CP+Hel-L, CP+Hel-H and Hel only groups were administered Hel via gavage at a dose of 2.55 mg/kg/day, 5.10 mg/kg/day, and 5.10 mg/kg/day once daily for 7 days. Mice in the Control and CP groups were administered the same volume of 0.5% CMC-Na aqueous solution. On the third day, 1 h after gavage, mice in the CP, CP+Hel-L and CP+Hel-H groups were intraperitoneally (*i.p*) injected with CP (20 mg/kg) to induce AKI. Mice in the Control group were *i.p.* given saline. Three days after the *i.p* injection of CP, the mice were fasted for 12 h and then euthanised. Mice were euthanized by intraperitoneal injection of 2% sodium pentobarbital (50 mg/kg).

**FIGURE 1:**
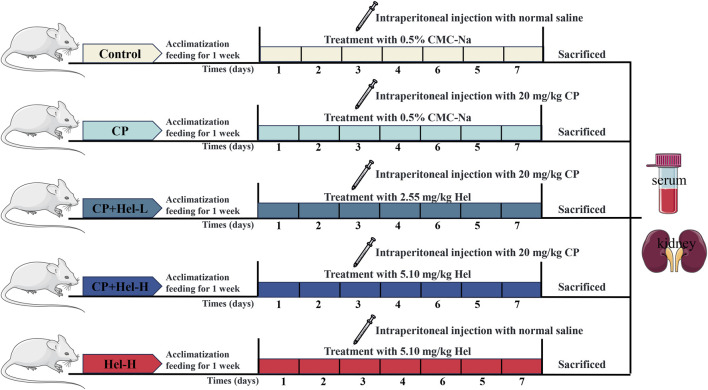
Experimental design of Hel treatment of CP-induced AKI.

Following ethical approval by the Animal Ethics Committee of Zunyi Medical University (Approval No. ZMU21-2304-009), the study was conducted. Harvested serum and kidney tissues were analyzed through multiple approaches, encompassing biochemical assays, histological evaluation, and metabolomic analysis. In order to evaluate kidney physiology, the kidney index was analyzed as a key parameter. The overall animal experimental design and drug intervention flowchart were shown in Figure 1.

$$\text{Kidney index}=\frac{\text{kidney}\;\text{weight}}{\text{body}\;\text{weight}}\times 100\%$$

### 2.4 Serum biochemistry indicator detection

Blood samples were drawn from the ocular venous plexus of mice, allowed to clot for 1 h, and then centrifuged (3,500 g, 10 min) to obtain serum. Kidney function was evaluated by measuring blood urea nitrogen (BUN) and serum creatinine (Scr) levels using an automated biochemistry analyzer (Beckman, USA).

### 2.5 Histological analysis

Kidney samples were fixed in 4% paraformaldehyde for 24 h, followed by dehydration and paraffin embedding. These tissues were sectioned into 4 μm-thick slices, stained with hematoxylin and eosin (H&E), and subsequently examined under an OLYMPUS BX43F optical microscope (Japan) at magnifications prior to photography.

### 2.6 Kidney metabolomics analysis

#### 2.6.1 Sample preparation and analytical conditions

Sample preparation commenced with the homogenization of 30 mg of thawed kidney samples using a pre-cooled grinder (30 Hz, 20 s). The resulting homogenate (20 mg) was then subjected to protein precipitation via addition of 400 μL of a methanol/water (7:3, v/v), followed by vortexing for 5 min. Following centrifugation (12,000 g, 10 min), 300 μL of the supernatant was collected and then recentrifuged. Finally, 200 μL of the clarified supernatant was dispensed into an injection vial for subsequent liquid chromatography (LC)-MS analysis.

Kidney metabolite analysis via UHPLC-MS was performed on a Shimadzu Nexera LC-30A system, with samples analyzed in both ESI+ and ESI- modes. An HSS T3 column (100 mm × 2.1 mm, 1.8 μm) was employed. Mobile phase A was 0.1% formic acid in water, and phase B was 0.1% formic acid in acetonitrile. The gradient elution program was set as: 5%–20% B (2 min), 20%–60% B (3 min), 60%–99% B (1 min, held for 1.5 min), 99%–5% B (0.1 min), and equilibration at 5% B (2.4 min). The injection volume was 4 μL, flow rate 0.4 mL/min, and column temperature maintained at 40 °C.

MS detection parameters were optimized to guarantee high-quality data collection. The ion source was operated with Gas 1 and Gas 2 at 50 psi, curtain gas at 25 psi, temperature at 550 °C, and de-clustering potential of ±60 V; floating ion spray voltage was set to 5,000 V and −4,000 V. Full-scan TOF-MS data were collected over 50–1,000 Da (accumulation time: 200 ms) with dynamic background subtraction. MS/MS product ion scans were performed over 25–1,000 Da (accumulation time: 40 ms) with collision energy of 30 eV and collision energy spread of 15 eV. Precursor ion selection criteria included unit resolution, charge state 1, intensity threshold of 100 cps, isotope exclusion within 4 Da, mass tolerance of 50 ppm, and a defined maximum number of candidate ions per cycle. Data acquisition employed the information-dependent acquisition (IDA) mode on the Analyst TF 1.7.1 software (Sciex, Canada).

#### 2.6.2 The validation of the analytical method

To minimize injection order-dependent bias, all kidney tissue extracts were analyzed via UHPLC-Q-TOF-MS in a randomized order. A pooled quality control (QC) sample (equal aliquots of all individual samples) was analyzed every 10 experimental samples during the run to monitor system stability, ensure data quality, and evaluate instrumental repeatability (n = 3). Wuhan Metware Biotechnology Co., Ltd. conducted the respective analyses.

#### 2.6.3 Metabolic data processing

The raw LC-MS data were converted to mzXML format using ProteoWizard software, followed by peak picking, alignment, and retention time calibration. Only metabolites with a coefficient of variation (CV) below 30% in QC samples were included in the subsequent statistical analysis and interpretation. The resulting high-quality dataset was then subjected to multivariate data analysis using MetaboAnalyst 6.0 (https://www.metaboanalyst.ca/). Principal component analysis (PCA), an unsupervised learning method, was employed to assess both intra-group variations and inter-group disparities across the dataset. Orthogonal partial least squares-discriminant analysis (OPLS-DA) was conducted to identify differential metabolites among groups. Differential metabolites were identified based on the following criteria: variable importance in the projection (VIP) > 1 from the OPLS-DA model, adjusted *P* < 0.05, and fold change (FC) > 2.5 or FC < 0.5. The significance of metabolites was determined based on p-values adjusted for multiple testing via the false discovery rate (FDR). Differential metabolites were identified and characterized by comparing the UPLC-MS data against entries in online databases, including HMDB (http://www.hmdb.ca), KEGG (http://www.kegg.com) and PubMed (https://pubmed.ncbi.nlm.nih.gov/). Key metabolic pathways were then enriched using MetaboAnalyst based on these annotated metabolites.

### 2.7 Statistical analysis

All data are presented as mean ± standard deviation (SD) and analyzed using SPSS 29.0. Data normality was examined by the Shapiro-Wilk test (Shaphiro and Wilk, 1965). Intergroup differences were assessed by one-way analysis of variance (ANOVA) after verifying variance homogeneity. Statistical significance was defined as *P* < 0.05 (marked as # or *) and high significance as *P* < 0.01 (marked as ## or **).

## 3 Results

### 3.1 The amino acid content of Hel

Previous compositional analyses have established that Hel is a chemically complex medicinal insect whose dry matter comprises lipids, proteins and amino acids, organic acids, and polysaccharides, together with minor amounts of minerals and other constituents (Liu, 2019). Against this background, the present study focused on characterizing the amino acid profile of the n-butanol extract used in the animal experiments, as amino acids represent a major bioactive-relevant fraction of the Hel protein content and can be quantified reproducibly by HPLC-MS/MS. A total of 21 amino acids were identified and quantified, comprising 9 essential, 9 non-essential, and 3 conditionally essential amino acids (Table 1).

### 3.2 Hel attenuated CP-induced AKI in mice

**FIGURE 2:**
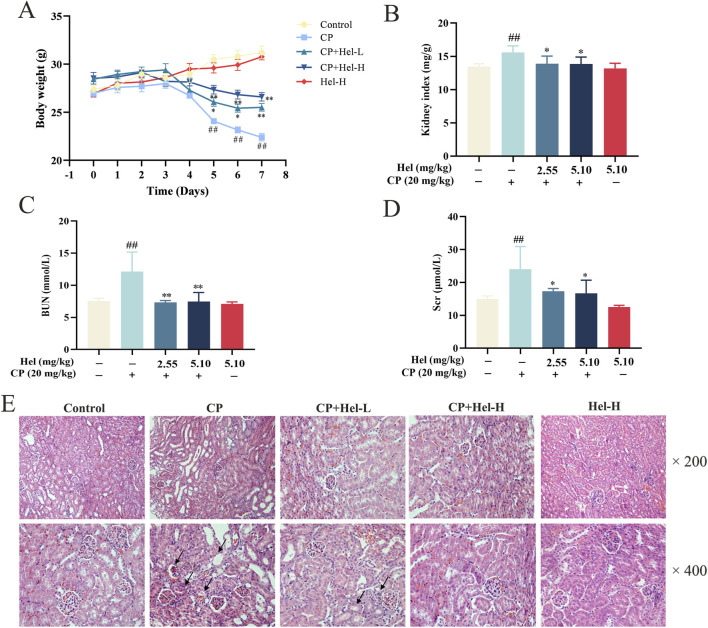
Hel improves CP-induced AKI *in vivo* (n = 6). Changes in the mouse body weight **(A)** and kidney index **(B)**. The levels of blood urea nitrogen (BUN) **(C)** and serum creatinine (Scr) **(D)** in the serum. Hematoxylin and eosin (H&E) staining was employed at magnification of ×200/400 **(E)** (mean ± SD, *P < 0.05 or **P < 0.01 vs. CP, #*P* < 0.05 or ##*P* < 0.01 vs. Control by One-way ANOVA).

For evaluating the protective efficacy of Hel in CP-induced AKI, we constructed mice with CP-induced AKI model, then administered Hel intervention, and finally quantified serum kidney function parameters. Compared to the Control group, CP challenge markedly elevated the levels of BUN and Scr, and the kidney index (Figures 2B–D) (*P* < 0.01), while also reducing the body weight of the mice (Figure 2A) (*P* < 0.01). Pretreatment with Hel at 5.10 mg/kg effectively reversed the CP-induced elevations in BUN, Scr, and kidney index, as well as restoring the reduced body weight (*P* < 0.05). Hel alone did not cause any significant alterations in BUN, Scr, or kidney index (Figures 2B–D).

### 3.3 Hel mitigates CP-induced kidney histopathology in mice

As shown in Figure 2E, H&E staining demonstrated that mice in both the Control group and the Hel-only administration group displayed normal renal morphological characteristics. In contrast, the CP group developed severe kidney injury, including glomerular atrophy, cytoplasmic vacuolation, inflammatory cell infiltration, and widespread tubular necrosis. Histological analysis revealed that kidney tissue damage was alleviated in the CP+Hel-L group compared with the CP group, and a more remarkable improvement was observed in the CP+Hel-H group. In conclusion, these findings demonstrate that Hel could improve kidney function and structural integrity in CP-induced AKI.

### 3.4 Metabolomics analysis

#### 3.4.1 Metabolic profile analysis

**FIGURE 3:**
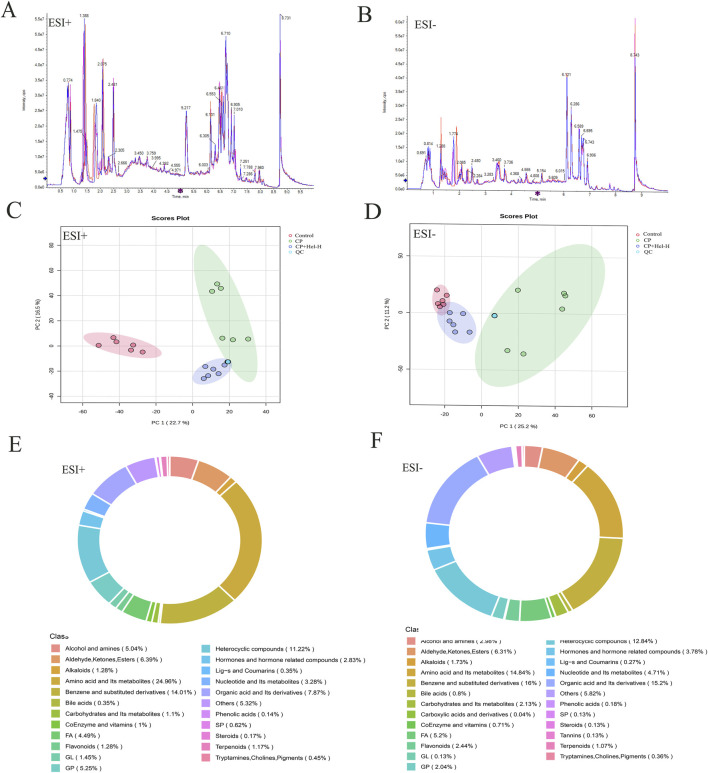
Discrimination of the kidney metabolic profile among Control, CP, CP+Hel-H mice (n = 6). Total ion current (TIC) of the kidney in ESI+ **(A)** and ESI- **(B)** ion modes. The score plots of PCA in ESI+ **(C)** and ESI- **(D)** ions modes. Superclass of identified metabolites of the kidney in ESI+ **(E)** and ESI- **(F)**.

To evaluate the instrumental repeatability and analytical stability, the total ion current (TIC) chromatograms were analyzed in both ESI+ and ESI- modes. The high degree of overlap in retention times and peak intensities across successive metabolite detections indicated excellent signal stability for the same samples analyzed at different time points (Figures 3A,B). Furthermore, the tight clustering of QC samples in PCA plots (Figures 3C,D) confirmed the excellent reproducibility and reliability of the metabolomics data acquisition.

To delineate the metabolic alterations induced by Hel intervention in the CP-induced AKI model, PCA was performed on the kidney metabolic profiles. The analysis revealed distinct separation among the Control, CP, and CP+Hel-H groups in both ionization modes (Figures 3C,D). Notably, the metabolic profile of the CP+Hel-H group exhibited a clear shift away from the CP group and trended towards the Control group. This trajectory indicates that Hel treatment effectively ameliorates CP-induced metabolic disruptions, driving a restoration of overall renal metabolic homeostasis. In addition, superclass classification of the detected metabolites identified amino acids, organic acids, and lipids as the predominant metabolite categories involved in this process (Figures 3E,F).

#### 3.4.2 Screen and identification of differential metabolites

**FIGURE 4:**
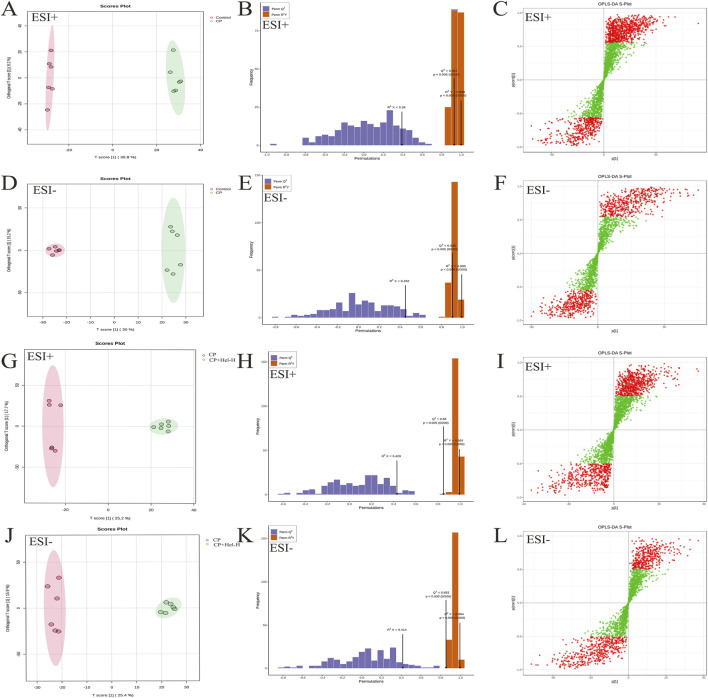
OPLS-DA analysis of Hel on CP-induced AKI (n = 6). The OPLS-DA analysis of the Control and CP groups in ESI+ **(A)** and ESI- **(D)** ion modes, respectively. 200 round permutation validation of the Control and CP group in ESI+ **(B)** and ESI- **(E)** ion modes, respectively. S-plots of the Control and the CP groups in ESI+ **(C)** and ESI- **(F)** ion modes, respectively. The OPLS-DA analysis of the CP and the CP+Hel-H group in ESI+ **(G)** and ESI- **(J)** ion modes, respectively. 200 times permutation test of the CP and the CP+Hel-H groups in ESI+ **(H)** and ESI- **(K)** ion modes, respectively. S-plots of the CP and the CP+Hel-H groups in ESI+ **(I)** and ESI- **(L)** ion modes, respectively.

OPLS-DA was employed to precisely screen for differential metabolites between the Control and CP groups (Figures 4A–F) as well as between the CP and CP+Hel-H groups (Figures 4G–L). Lower R^2^ and Q^2^ values for the permuted data compared to the original models, together with a negative Q^2^ intercept in the 200-permutation test, validate the robustness and predictive capability of the OPLS-DA models.

**FIGURE 5:**
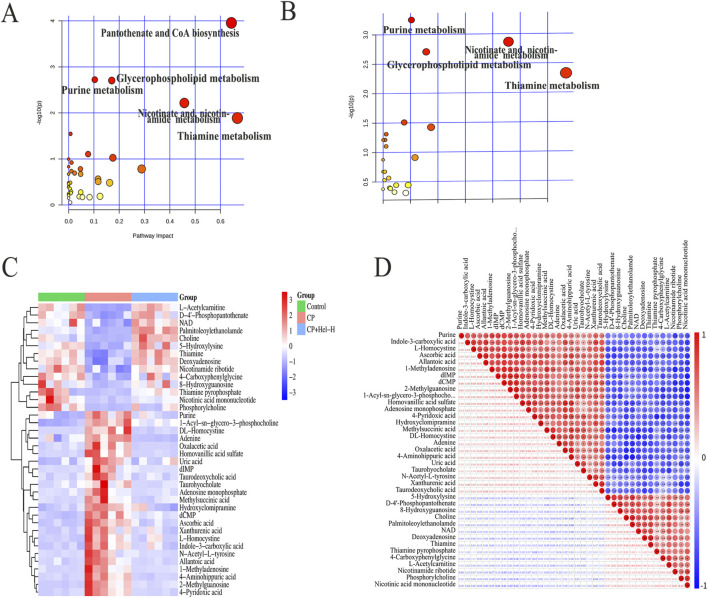
Kidney metabolism analysis of Hel in CP-induced AKI. The disorder metabolic pathways in the kidney of AKI mice **(A)**. The disorder metabolic pathways were corrected by Hel in the kidney of AKI mice **(B)**. The heatmap of 38 biomarkers metabolites **(C)**. Correlation analysis of differential metabolites **(D)**.

A total of 83 differential metabolites were identified between the Control and CP groups, and changes their relative abundance changes were subsequently analyzed. After Hel-H intervention, 38 differential metabolites were significantly partly restored to normal levels, which is depicted in the heatmap (Figure 5C). The characteristics of these significantly altered and partially restored differential metabolites are presented in Table 2. The findings indicate that Hel treatment could partially reverse the disruption of kidney differential metabolites induced by CP, thereby alleviating CP-induced AKI.

**TABLE 2:** 38 identified potential differential metabolites regulated by Hel.

No	Metabolites	Formula	Adduct	KEGG ID	Molecular weight (Da)	Rt (min)	VIP		FC	
							CP vs. control	Hel vs. CP	CP vs. control	Hel vs. CP
1	Choline	C_5_H_12_N+	[M+]+	C00114	86.10	1.00	1.43	1.00	0.09	26.45
2	Purine	C_5_H_4_N_4_	[M+H]+	C15587	120.04	1.77	1.43	1.63	2.55	0.48
3	Oxalacetic acid	C_4_H_4_O_5_	[2M-H]-	C00036	132.01	3.41	1.92	2.31	7.71	0.07
4	Methylsuccinic acid	C_5_H_8_O_4_	[M-H]-	C02726	132.04	2.35	1.34	1.39	2.92	0.35
5	Adenine	C_5_H_5_N_5_	[M+H]+	C00147	135.06	2.37	1.68	1.55	2.50	0.23
6	Indole-3-carboxylic acid	C_9_H_7_NO_2_	[M-H]-	C19837	161.05	2.86	1.63	1.37	4.03	0.38
7	5-Hydroxylysine	C_6_H_14_N_2_O_3_	[M-H]-	C16741	162.10	0.64	1.74	2.00	0.21	6.43
8	Uric acid	C_5_H_4_N_4_O_3_	[M+H]+	C00366	168.03	1.40	1.52	1.26	2.95	0.50
9	Ascorbic acid	C_6_H_8_O_6_	[2M-H]-	C00072	176.03	0.89	1.64	1.55	3.98	0.33
10	Allantoic acid	C_4_H_8_N_4_O_4_	[M-H2O-H]-	C00499	176.06	0.85	1.31	1.39	2.66	0.38
11	4-Pyridoxic acid	C_8_H_9_NO_4_	[M-H]-	C00847	183.05	1.94	1.88	1.92	6.34	0.17
12	Phosphorylcholine	C_5_H_15_NO_4_P+	[M+Na]+	C00588	184.07	1.50	2.20	2.35	0.13	3.75
13	4-Aminohippuric acid	C_9_H_10_N_2_O_3_	[M-H]-	C01029	194.07	3.12	1.94	2.25	7.13	0.10
14	4-Carboxyphenylglycine	C_9_H_9_NO_4_	[2M-H]-	C20678	195.05	1.44	1.68	1.38	0.24	3.17
15	L-Acetylcarnitine	C_9_H_17_NO_4_	[M+H]+	C02571	203.12	0.88	1.95	2.22	0.20	5.22
16	Xanthurenic acid	C_10_H_7_NO_4_	[M+H]+	C02470	205.04	2.86	1.65	1.54	3.91	0.36
17	N-Acetyl-L-tyrosine	C_11_H_13_NO_4_	[M-H]-	C01657	223.09	3.06	1.84	1.86	10.40	0.12
18	Deoxyadenosine	C_10_H_13_N_5_O_3_	[M+F]-	C00559	251.10	1.90	1.27	1.44	0.45	2.53
19	Homovanillic acid sulfate	C_9_H_10_O_7_S	[M-H]-	C05582	262.02	3.41	2.06	2.15	10.23	0.10
20	Thiamine	C_12_H_17_N_4_OS+	[M+CH3COO]-	C00378	265.11	2.51	1.43	1.64	0.38	3.13
21	DL-Homocystine	C_8_H_16_N_2_O_4_S_2_	[M+H-H2O]+	C01817	268.06	3.03	2.14	2.65	8.26	0.09
22	L-Homocystine	C_8_H_16_N_2_O_4_S_2_	[M+Cl]-	C01817	268.06	1.91	1.75	1.42	5.47	0.35
23	1-Methyladenosine	C_11_H_15_N_5_O_4_	[M+H]+	C02494	281.11	1.38	1.84	2.01	5.53	0.21
24	D-4′-Phosphopantothenate	C_9_H_15_NO_8_P-_3_	[2M-H]-	C03492	296.05	2.03	1.37	1.50	0.34	3.02
25	2-Methylguanosine	C_11_H_15_N_5_O_5_	[M-H]-	C04545	297.11	2.06	1.69	1.48	4.50	0.33
26	Palmitoleoylethanolamde	C_18_H_35_NO_2_	[M+H]+	—	297.27	5.68	1.68	1.37	0.50	3.89
27	8-Hydroxyguanosine	C_10_H_13_N_5_O_6_	[M-H]-	—	299.09	1.46	1.29	1.39	0.29	3.04
28	dCMP	C_9_H_14_N_3_O_7_P	[M+NH4]+	C00239	307.06	2.62	1.70	1.44	3.68	0.43
29	dIMP	C_10_H_13_N_4_O_7_P	[M-]-	C06196	332.05	3.30	2.55	2.07	48.67	0.08
30	Nicotinamide ribotide	C_11_H_15_N_2_O_8_P	[M+H]+	C00455	334.06	0.84	1.57	1.17	0.35	2.51
31	Nicotinic acid mononucleotide	C_11_H_15_NO_9_P+	[M+CH3COO]-	C01185	336.05	2.62	1.84	1.76	0.15	2.62
32	Thiamine pyrophosphate	C_12_H_18_N_4_O_4_PS+	[M-]-	C01081	345.08	2.31	1.40	1.51	0.26	2.76
33	Adenosine monophosphate	C_10_H_14_N_5_O_7_P	[M-H]-	C00946	347.06	1.83	1.86	1.99	8.18	0.11
34	1-Acyl-sn-glycero-3-phosphocholine	C_23_H_48_NO_7_P	[M+H-H2O]+	C00157	481.32	7.38	2.05	1.05	5.37	0.50
35	Taurodeoxycholic acid	C_26_H_45_NO_6_S	[M-H]-	C05463	499.30	7.85	1.67	1.82	5.55	0.18
36	Taurohyocholate	C_26_H_45_NO_7_S	[M+NH4]+	C15516	515.29	6.60	1.74	2.14	4.96	0.15
37	Hydroxyclomipramine	C_41_H_72_O_5_	[M+Na]+	C00165	644.54	8.77	1.55	1.41	2.88	0.43
38	NAD	C_21_H_28_N_7_O_14_P_2_+	[M-H]-	C00003	664.12	1.44	1.20	1.16	0.43	2.59

#### 3.4.3 Metabolic pathway analysis and potential biomarker identification

**FIGURE 6:**
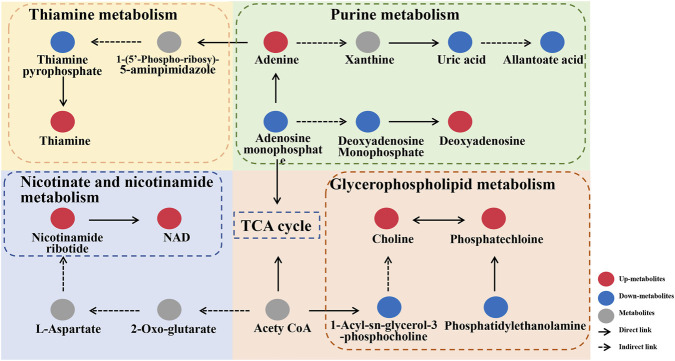
Integrated analysis of brief metabolic pathways (The red represents upregulated metabolites by Hel while the blue represents downregulated metabolites by Hel).

**FIGURE 7:**
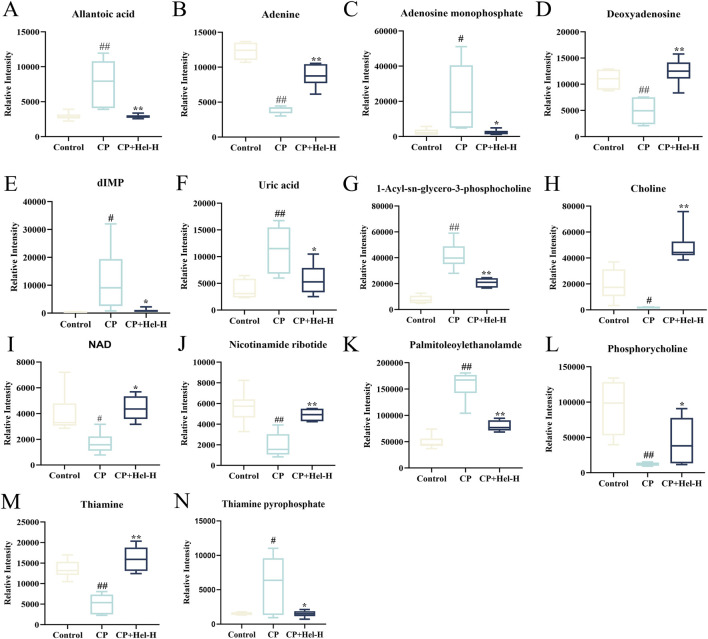
Potential metabolic biomarkers change of Hel on CP-induced AKI. Allantonic acid **(A)**. Adenine **(B)**. Adenosine monophospahte **(C)**. Deoxyadenosine **(D)**. dIMP **(E)**. Uric acid **(F)**. 1-Acyl-sn-glycero-3-phosphocholine **(G)**. Choline **(H)**. NAD **(I)**. Nicotinamide ribotide **(J)**. Palmitoleoylethanolamde **(K)**. Phosphorycholine **(L)**. Thiamine **(M)**. Thiamine pyrophosphate **(N)**. (mean ± SD, *P < 0.05 or **P < 0.01 vs. CP, #*P* < 0.05 or ##*P* < 0.01 vs. Control by One-way ANOVA).

To further reveal the therapeutic mechanism of Hel, pathway enrichment analysis was conducted on differential metabolites using the KEGG database via MetaboAnalyst 6.0, where impact values >0.1 and *P* < 0.05 were used as screening criteria. As shown in Figure 5A, CP mainly affected five metabolic pathways, including purine metabolism, pantothenate and CoA biosynthesis, glycerophospholipid metabolism, thiamine metabolism, and nicotinate and nicotinamide metabolism. Hel-H treatment ameliorated CP-induced AKI by reshaping key metabolic pathways in the kidney. The protective effect was particularly mediated by significant modulation of purine metabolism, thiamine metabolism, glycerophospholipid metabolism, and nicotinate nicotinamide metabolism (Figure 5; Table 3). Correlation analysis revealed that the metabolites indicated by red nodes were mostly positively correlated, purine, uric acid and 1-Acyl-sn-glycero-3-phosphocholine (LPC) were positively correlated with 24 metabolites (Figure 5D). The integrated metabolic pathways were presented (Figure 6), highlighting 14 identified differential metabolites involved in these pathways, as illustrated in Figure 7.

**TABLE 3:** Metabolism pathway disorders in mice kidney regulated by Hel.

Term	Overlap	P-value	Impact value	Hits compound
Purine metabolism	6/71	0.000189	0.10326	Allantoic acid, adenine, dIMP, deoxyadenosine, uric acid, and adenosine monophosphate
Nicotinate and nicotinamide metabolism	3/15	0.000792	0.45702	Nicotinamide ribotide, NAD, and nicotinic acid mononucleotide
Glycerophospholipid metabolism	4/36	0.000965	0.15704	Palmitoleoylethanolamde, phosphorylcholine, choline, and 1-acyl-sn-glycero-3-phosphocholine
Thiamine metabolism	2/7	0.003292	0.66667	Thiamine and thiamine pyrophosphate

## 4 Discussion

CP is a first-line chemotherapeutic agent for various cancers, nevertheless, its utility is significantly constrained by dose-limiting nephrotoxicity, which manifests as AKI (Dasari and Tchounwou, 2014; Sears et al., 2023). Consequently, preventing CP-induced AKI has become crucial for enhancing therapeutic efficacy, prompting extensive research (Lu et al., 2023; Xu et al., 2024). TCM has been practiced in China for thousands of years, and its use in preventing and managing chemotherapy-related adverse reactions, including CP-induced AKI, has been extensively investigated (Guo et al., 2025; Xu et al., 2019). As a traditional edible insect with exceptional nutritional value, Hel has been traditionally used as a functional food for managing kidney-related dysfunctions (Shi et al., 2019). Thus, this study employed a combination of metabolomics and metabolite network analysis to evaluate the nutritional effects and metabolic regulatory potential of Hel-derived protein, aiming to provide scientific evidence for its application as a high-value alternative protein source in insect nutrition research.

### 4.1 Hel significantly improves CP-induced AKI

To evaluate the nephroprotective effect of Hel against CP-induced AKI, mice were pretreated orally with Hel for 7 consecutive days, with a single injection of CP administered on the third day. CP challenge induced kidney insufficiency, as indicated by elevated serum levels of kidney injury biomarkers such as Scr and BUN. These changes were significantly attenuated by Hel pretreatment. H&E staining also confirmed that CP injection induced marked kidney damage, characterized by tubular necrosis, protein casts, and inflammatory cell infiltration, consistent with prior reports (Xu et al., 2023). Hel pretreatment attenuated the symptoms and markedly alleviated CP-induced AKI, demonstrating a dose-dependent decrease in kidney index along with an increase in body weight. Hence, Hel pretreatment improved CP-induced AKI, exerting a nephroprotective effect.

### 4.2 Hel-improved CP-induced AKI biometabolic mechanisms

Subsequently, untargeted metabolomic profiling was performed to characterize the metabolic perturbations induced by CP and regulated by Hel. Notable differences in metabolic profiles existed among the Control, CP, and CP+Hel-H groups, while the CP+Hel-H group closely paralleled the Control group, suggesting that Hel exerted a substantial regulatory effect on the kidney metabolomic profile. This analysis led to the identification of 38 differential metabolites. Pathway enrichment analysis implicated these metabolites in four key metabolic pathways: glycerophospholipid metabolism, purine metabolism, nicotinate and nicotinamide metabolism, and thiamine metabolism.

#### 4.2.1 Glycerophospholipid metabolism

Palmitoleoylethanolamide (POEA), phosphorylcholine (PC), choline, and LPC were identified as key biomarkers associated with glycerophospholipid metabolism, which is critical for maintaining membrane integrity and generating lipid mediators that regulate inflammation and immune responses (Huang et al., 2012; Li et al., 2021; Sonkar et al., 2019). Evidence suggests that sustained inflammation and oxidative stress are key drivers of AKI (Wang et al., 2024). Specifically, PC serves as a major structural component of biological membranes and participates in lipoprotein assembly (Shen et al., 2023). Through the action of cholesterol acyltransferases, PC can be metabolized to LPC, which has been shown to promote ATP release (Gallazzini and Burg, 2009; Ismaeel and Qadri, 2021). Elevated urinary LPC levels in patients with diabetic nephropathy implicates this lipid metabolite in renal inflammation and fibrosis (Saulnier-Blache et al., 2017). In contrast, POEA exerts anti-inflammatory effects by suppressing the expression of IL-6 and TNF-α in high-fat diet (HFD) rats (Tovar et al., 2021). In our CP-induced AKI model, kidney LPC levels increased, a change reversed by Hel pretreatment. Conversely, POEA has demonstrated anti-inflammatory effects. These findings suggest that Hel alleviates AKI, at least in part, by reducing LPC to mitigate inflammatory processes.

#### 4.2.2 Purine metabolism

Key purine metabolites, including allantoic acid, adenine, deoxyinosine monophosphate (dIMP), deoxyadenosine, uric acid, and AMP, were altered in our model. Purine metabolism is fundamental to key biological processes, including nucleic acid synthesis, cellular energy transfer, and the modulation of signaling networks and metabolic balance (Tran et al., 2024). During ATP synthesis, AMP is converted into IMP in cells, and functions primarily as an intermediate in energy metabolism (Zabielska et al., 2015). Purine metabolites, especially uric acid, which has been reported to be elevated in both clinical and animal studies, can trigger inflammation and oxidative stress (Liu et al., 2021; Zhou et al., 2023). For instance, flavonoid extracts from Poria cocos rhizomes significantly reduce the expression of inflammatory mediators and oxidative stress markers in renal tissue from rat models of uric acid nephropathy (Wang et al., 2019). Inhibition of adenine accumulation reduces renal hypertrophy and kidney injury in diabetic mice (Sharma et al., 2023). In our study, CP-induced AKI led to decreased AMP and deoxyadenosine, but increased uric acid, adenine, and allantoic acid, indicating disrupted energy metabolism and elevated pro-inflammatory. Hel pretreatment normalized these metabolite levels, suggesting its nephroprotective effect involves restoring purine metabolism homeostasis.

#### 4.2.3 Nicotinate and nicotinamide metabolism

Nicotinamide ribotide and NAD were key altered intermediates in nicotinate and nicotinamide metabolism. Nicotinate and nicotinamide metabolism modulates DNA repair, energy metabolism, and intracellular signal transduction (Braidy et al., 2019; Nikas et al., 2020). Maintenance NAD homeostasis enhances resistance to infections and inflammatory diseases and is closely linked to the prevention of kidney disease progression (Amjad et al., 2021; Covarrubias et al., 2021). Multiple investigations have indicated that NAD concentrations are substantially reduced in patients and mice affected by AKI and CKD increased intracellular ROS levels and impaired mitochondrial function (Ix et al., 2019; Zheng et al., 2019). Furthermore, research has demonstrated that NAD serves as a potential biomarker for kidney injury, while therapeutic interventions can alleviate infection and inflammation by increasing NAD levels (Bignon et al., 2022). Our results align with this, showing decreased nicotinamide ribotide and NAD in AKI mice, which Hel pretreatment could reversed, thereby reducing kidney injury by suppressing inflammation.

#### 4.2.4 Thiamine metabolism

Thiamine (vitamin B_1_) and its active form, thiamine pyrophosphate (TTP), are essential for human health, functioning as a coenzyme in fundamental metabolic pathways including the carbohydrate metabolism and pentose phosphate pathway (Bozic and Lavrnja, 2023). As a coenzyme in these basic metabolisms, its deficiency can cause disturbances in energy metabolism and oxidative stress (Kartal and Palabiyik, 2019; Moradi and Said, 2016). Research has shown that thiamine supplementation attenuates the inflammatory response and mitigates tissue impairment in mice with sepsis-induced AKI through reducing proinflammatory cytokine IL-6 and chemokines KC and MIP-2 (Kim et al., 2022). Our study found that TTP levels were diminished in CP-induced AKI mice, and Hel pretreatment upregulation TTP. These results indicate that Hel may regulate thiamine metabolism disorders by elevating TTP levels, and consequently alleviate kidney injury through improving energy metabolism and suppressing oxidative stress.

However, this study has several limitations. First, while *in vivo* efficacy was validated across multiple doses, comprehensive metabolomics profiling was restricted to the optimal dose (CP+Hel-H), precluding the establishment of a complete dose-dependent metabolic trajectory. Second, our mechanistic insights are derived exclusively from an *in vivo* murine model; without parallel *in vitro* validation, it remains challenging to distinguish whether the observed renoprotection is a direct tubular effect or a secondary systemic response, which inherently limits direct clinical translation. Finally, as the Hel preparation is a chemically complex extract, the specific bioactive constituents responsible for its efficacy remain unidentified, thereby constraining targeted target validation and pharmacokinetic analyses. Future investigations incorporating purified active fractions, robust *in vitro* models, and multi-dose omics designs are warranted to fully elucidate its translational potential.

## 5 Conclusion

In conclusion, this study integrates *in vivo* pharmacological evaluation with comprehensive metabolomics to elucidate the protective effects and underlying metabolic mechanisms of Hel against CP-induced AKI. Our findings demonstrate that Hel treatment effectively ameliorates renal injury by restoring metabolic homeostasis, significantly reversing the aberrant levels of 38 key metabolites. Mechanistically, these restorative effects are primarily driven by the modulation of critical pathways, including glycerophospholipid, purine, nicotinate and nicotinamide, and thiamine metabolism. Ultimately, this metabolomics-driven approach not only highlights the therapeutic potential of Hel in mitigating drug-induced nephrotoxicity but also provides a robust foundation for future strategies targeting metabolic reprogramming in kidney diseases.

## Data Availability

The raw data supporting the conclusions of this article will be made available by the authors, without undue reservation.
